# The first revision of the carnivorous land snail family Streptaxidae in Laos, with description of three new species (Pulmonata, Stylommatophora, Streptaxidae)

**DOI:** 10.3897/zookeys.589.7933

**Published:** 2016-05-16

**Authors:** Khamla Inkhavilay, Thanit Siriboon, Chirasak Sutcharit, Ben Rowson, Somsak Panha

**Affiliations:** 1 Animal Systematics Research Unit, Department of Biology, Faculty of Science, Chulalongkorn University, Bangkok 10330, Thailand; 2 Department of Natural Sciences, National Museum of Wales, Cathays Park, Cardiff CF10 3NP, United Kingdom

**Keywords:** Limestone, tropical forest, systematics, type specimen, Southeast Asia, predator, taxonomy, aphally

## Abstract

The family Streptaxidae in Laos is revised. Twelve species are known, mainly from limestone areas, in the genera *Discartemon* Pfeiffer, 1856, *Perrottetia* Kobelt, 1905, *Haploptychius* Möllendorff, 1906, and *Indoartemon* Forcart, 1946. Three new species, *Perrottetia unidentata* **sp. n.** and *Perrottetia megadentata* **sp. n.** from northern and central Laos, and *Indoartemon diodonta* **sp. n.** from central Laos, are described. All eight species of these three genera previously recorded from Laos are revised and discussed based on examined material from Laos, Cambodia, Vietnam and Thailand. Type material was examined and lectotypes are designated. Details of genital anatomy and radulae are provided, including the first detailed genitalia and radula descriptions from *Haploptychius*. Two novelties in Streptaxidae, a vaginal caecum, and the occurrence of aphallic individuals, are reported from *Haploptychius pellucens* (Pfeiffer, 1863).

## Introduction

The Streptaxoidea currently comprises two families, the worldwide Streptaxidae Gray, 1860 and the Southeast Asian endemic Diapheridae Panha and Naggs, 2010 (Richardson 1988, Schileyko 2000, Rowson et al 2010a, Sutcharit et al. 2010). The Streptaxidae Gray, 1860 are active predators with an eccentric to cylindrical shell, usually with apertural dentition, and a yellowish to orange soft body (Zilch 1960, Schileyko 2000, Rowson et al. 2010b, Siriboon et al 2013, 2014a, b).

Early classifications of the family such as W. Kobelt (1905–6), used mainly shell shape and the arrangement of apertural dentition. However, many shell characters are highly conserved or occur recurrently, making some species and genera difficult to separate. Fortunately, the reproductive organs of streptaxids can also be taxonomically significant (e.g. Schileyko 2000, Rowson and Tattersfield 2013, Siriboon et al. 2013, 2014a, b). Few reports have contributed data on the genitalia of Southeast Asian taxa (e.g. Stoliczka 1871, Berry 1963, 1965) until recently (Siriboon et al. 2013, 2014a, b, Páll-Gergely et al. 2015).

In Indochina, streptaxid diversity was throught to comprise only 10 genera and about 40 species (Bruggen 1967). However, in the last decade 21 new species (more than half the previous total) and one new genus have been described from Indochina (Siriboon et al. 2013, 2014a, b, Do and Do 2015). Thirty-seven species are recorded from Thailand (Panha 1996, Hemmen and Hemmen 2001, Siriboon et al. 2013, 2014a, b), 10 from Myanmar (Blanford and Godwin-Austen 1908), and 45 from Vietnam (Schileyko 2011). In contrast, only three species were reported from Laos in the past two centuries (Pfeiffer 1863, Möllendorff 1898), with three others added in recent years (Schileyko 2011, Do and Do 2015).

Almost all groups of the land snail fauna in Laos have been less-well studied than those of neighbouring areas. The Lao People’s Democratic Republic, until recently encompassed some of the most significant forest areas remaining in Southeast Asia such as mountainous areas in the north and limestone karsts in central area, and some of the most intact biota left in Asia (Kemp 2011). Those habitat characteristics also harbor diverse of terrestrial molluscan fauna. The present paper focuses on the four genera, *Discartemon* Pfeiffer, 1856, *Perrottetia* Kobelt, 1905, *Haploptychius* Möllendorff, 1906, and *Indoartemon* Forcart, 1946 that were formerly recorded from Laos. Genital anatomy and shell micro-structures of are carefully investigated. The type specimens of all known species were examined, and the penial hooks and radula morphology of *Haploptychius* are defined for the first time. This adds significantly to knowledge of the Streptaxidae in Indochina and especially in Laos.

## Materials and methods

Animals were collected from evergreen forest in the north, and limestone karsts and dipterocarp forest in the south of Laos. Live specimens were photographed and then stored at -20 °C and then preserved in 70% ethanol (v/v) for anatomical studies. The identifications were based on Pfeiffer (1863), Möllendorff (1898), Kobelt (1905–1906), Blanford and Godwin-Austen (1908), Bavay and Dautzenberg (1903, 1908), and Siriboon et al. (2013, 2014b). Shell height (H), shell width (W), whorl count and H/W ratio were measured and interpreted following Siriboon et al. (2013). Shells and genitalia were investigated and digital images taken using Cell’D Imaging Software. All live adult specimens of each species were dissected and the genitalia examined under a stereo-microscope and representatives selected for illustrations under a camera lucida. The buccal masses were removed, and the radulae were soaked in 10% NaOH then cleaned in distilled water. Radula, penial hooks and vaginal hooks were examined and photographed under SEM (JEOL, JSM-5410 LV). In the descriptions, ‘proximal’ relates to the genital orifice, and ‘distal’ refers to the region furthest away from the genital orifice. The term ‘vaginal caecum’ is defined herein.

**Anatomical abbreviations.** ag, albumen gland; at, atrium; fo, free oviduct; gd, gametolytic duct; gs, gametolytic sac; hd, hermaphroditic duct; ov, oviduct; p, penis; pr, penial retractor muscle; ps, penial sheath; psr, penial sheath retractor muscle; sv, seminal vesicle; ta, talon; v, vagina; vc, vaginal caecum; vd, vas deferens (Sutcharit et al. 2010, Siriboon et al. 2013, 2014a, b).

**Institutional abbreviations.** Materials examined in this study were deposited in the following institutions:

CUMZ
Chulalongkorn University Museum of Zoology, Bangkok 


MNHN
Muséum National ďHistoire Naturelle, Paris 


NHMUK
 The Natural History Museum, London



NUOL
National University of Laos, Vientiane 


SMF
 Forschungsinstitut und Naturmuseum Senckenberg, Frankfurt am Main 


## Systematics

### Family Streptaxidae Gray, 1860

#### Genus Discartemon

Taxon classificationAnimaliaPulmonataStreptaxidae

Pfeiffer, 1856

Discartemon Pfeiffer, 1856: 173. Siriboon et al. 2014a: 48, 49.
Odontartemon (Discartemon) —Kobelt 1905: 91, 96.

##### Type species.

*Streptaxis discus* Pfeiffer, 1851, by subsequent designation by Ancey (1884: 399).

##### Remark.

The genus was recently revised. For complete illustrations, species descriptions and dichotomous key see Siriboon et al. (2014a).

#### Discartemon discus

Taxon classificationAnimaliaPulmonataStreptaxidae

(Pfeiffer, 1853)

Streptaxis discus Pfeiffer, 1853: 252. Type locality: Unknown.
Streptaxis (Discartemon) paradiscus Möllendorff, 1900: 117. Type locality: Phucson bei Touranne, Annam [Da Nang Province, Vietnam].
Discartemon discus —Siriboon et al. 2014a: 53–55, figs 4a–c, 11a–c, 22a.

##### Material examined.

Lectotype of *Streptaxis discus* Pfeiffer, 1853 NHMUK 20130684. Lectotype of *Streptaxis paradiscus* Möllendorff, 1900 SMF 108534 and paralectotypes SMF 108535 (5 shells).

##### Remarks.

*Discartemon discus* has been recently re-described from the shell, genitalia and radula, and type specimens were re-investigated and illustrated (see Siriboon et al. 2014a).

All previous records of this species were all from “Annam” (Siriboon et al. 2014a). This term is a historical political division during the colonial period, with an uncertain boundary. The distribution of *Discartemon discus* (=*Discartemon paradiscus*) in Laos was reported by Schileyko (2000: 784, 2011: 23). However, no specimens were found by the present study and the records from Laos remain to be confirmed.

#### Genus Haploptychius

Taxon classificationAnimaliaPulmonataStreptaxidae

Möllendorff, 1906

Haploptychius Möllendorff in Kobelt 1906: 127. Zilch 1960: 562. Richardson 1988: 211. Schileyko 2000: 796, 797.
Odontartemon (Haploptychius) —Thiele 1931: 730. Forcart 1946: 215.
Oophana (Haploptychius) —Benthem Jutting 1954: 76, 95.

##### Type species.

*Streptaxis sinensis* Gould, 1859, by original designation.

##### Description.

Shell depressed to very distorted, mostly white-hyaline or transparent. Shell surface smooth and glossy or with fine radial ridges. Embryonic shell smooth; following whorls increasing regularly; penultimate whorls slightly to strongly extended beyond body whorl. Last whorl rounded and more or less deviated from the vertical axis. Umbilicus narrowly open and deep. Aperture sub-circular to semi-ovate. Peristome expanded and reflected. Apertural dentition always consisting of a single parietal lamella. Schileyko (2000) includes species with a “smooth” parietal wall, i.e. without a lamella in *Haploptychius*, but whether such taxa belong in this genus requires further investigation.

Live specimens exhibit a semi-transparent bright yellow body, sometimes with brownish spots; skin reticulated. Upper tentacles yellow to orange, long, with black eye-spot on tip; lower tentacles short. Brownish digestive gland and black kidney may be visible through transparent shell. Foot narrow, undivided and with short tail.

Genitalia with long and slender penis; penial sheath long, about a half to whole length of penis. Internal wall of penis with numerous long and slender penial hooks in longitudinal arrangement. Vas deferens passes under penial sheath before connecting apically to penis. Vagina and free oviduct short. Seminal vesicle present, convoluted and short. Vaginal hooks not found.

##### Remarks.

Currently, the genus *Haploptychius* consists of about 40 nominal species distributed from India to Indochina, south of China and Greater Sunda Islands (Kobelt 1906, Zilch 1961, Richardson 1988, Schileyko 2000). Fifteen species were reported from Indochina, of which only three species: *Haploptychius pellucens* (Pfeiffer, 1863), *Haploptychius porrectus* (Pfeiffer, 1863) and *Haploptychius fischeri* (Morlet, 1887) were recorded from Laos (see Gude 1903, Kobelt 1906, Schileyko 2011).

General shell morphology of *Haploptychius* is quite similar to *Oophana* Ancey, 1884 and *Indoartemon* Forcast, 1946. However, it differs in having only a parietal lamella; while *Oophana* usually has parietal, palatal, columellar and basal lamellae, and *Indoartemon* always has parietal and basal lamellae. In addition, the genitalia of *Haploptychius* have a penial sheath extends about a half to entire the penis length, vas deferens passes through penial sheath, and long slender penial hooks. In *Oophana*, the vas deferens enter the penial sheath apically with very short vagina (Berry 1963, Schileyko 2000); and *Indoartemon*, the vas deferens attached (not pass through) the penial sheath, with small and short penial hooks (Siriboon et al. 2013).

*Carinartemis* Siriboon & Panha, 2013 resembles *Haploptychius* in having only a parietal lamella. However, it differs from *Haploptychius* in its very sharp peripheral keel and having the last whorl more deviated from the vertical axis. In addition, the genitalia has thick or thin penial sheath, penial hook short and stout, and vaginal hooks present (Siriboon et al. 2014b).

The relatively large, distorted heliciform shell and dentition restricted to a parietal lamella clearly differentiate *Haploptychius* from *Discartemon* Pfeiffer, 1856 and *Perrottetia* Kobelt, 1905 (Schileyko 2000, Siriboon et al. 2013, 2014a, b).

#### Haploptychius pellucens

Taxon classificationAnimaliaPulmonataStreptaxidae

(Pfeiffer, 1863)

**Figure 1.**
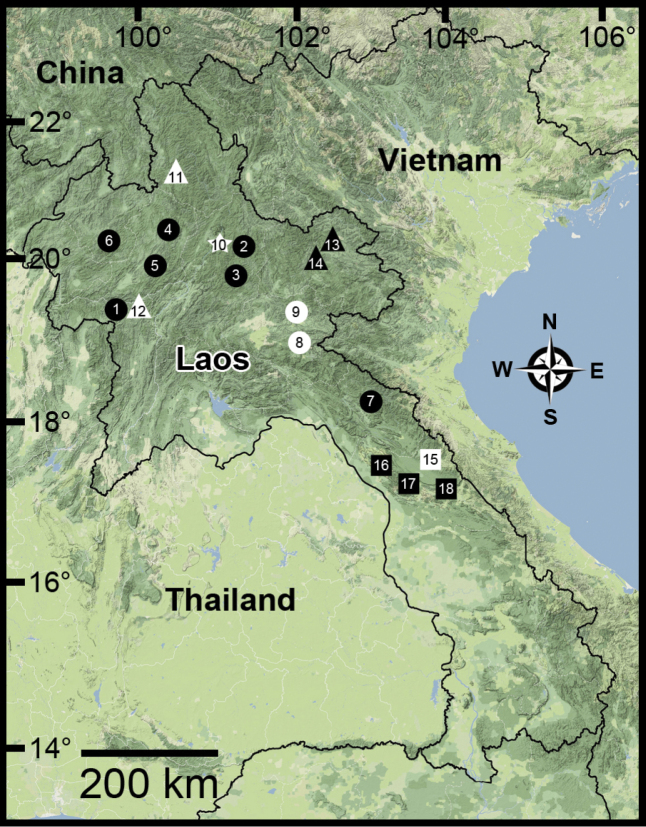
Approximate locations of the type localities of *Haploptychius* spp., *Perrottetia* spp., and *Indoartemon* spp. in Laos. Described species (●) *Haploptychius pellucens*, (○) *Haploptychius porrectus*, (★) *Haploptychius blaisei*, (△) *Perrotettia aquilonaria*, (▲) *Perrottetia unidentata* sp. n., (□) *Perrottetia megadentata* sp. n. and (■) *Indoartemon diodonta* sp. n. The numbered localities are detailed in Table 1, except locality no. 6 is from Tam Kao Rao, Vieng Phoukha, Luang Namtha, Laos, and no. 11 from Ban Bo Khoun, Boun Neua, Phongsaly, Laos.

**Figure 2.**
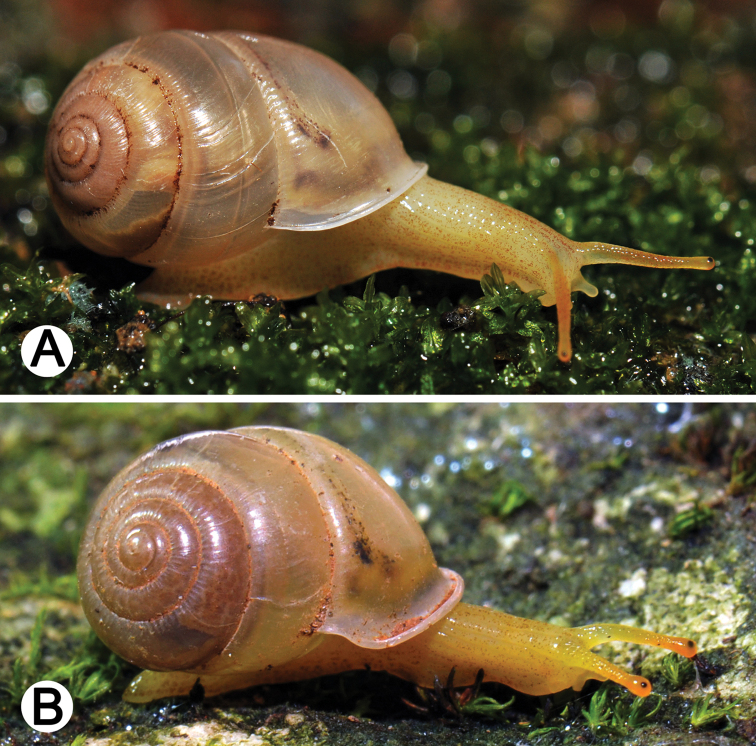
Living snails of **A** *Haploptychius pellucens* CUMZ 6265, from Xayabouly (shell width about 11 mm) and **B** *Haploptychius porrectus* CUMZ 6273, from Xieng Khuang (shell width about 7 mm).

**Figure 3.**
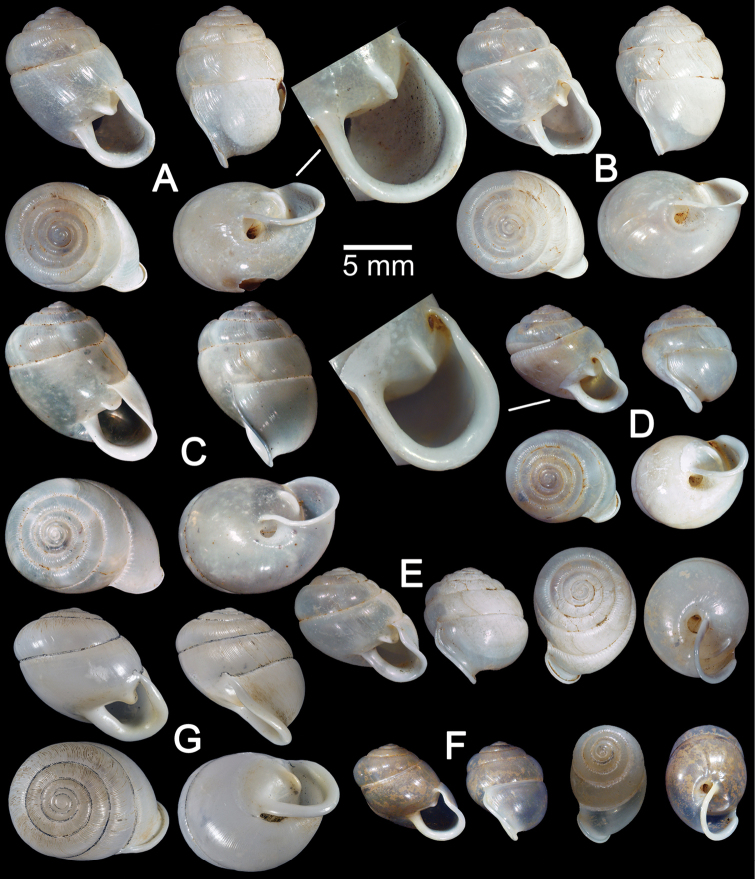
Shells of *Haploptychius* spp. **A–C** *Haploptychius pellucens* **A** lectotype NHMUK 20160249.1 with apertural dentition **B** paralectotype NHMUK 20160249.2, and **C** specimen CUMZ 6264, from Xayabouly **D–F** *Haploptychius porrectus* **D** lectotype NHMUK 20140750.1 **E** paralectotype NHMUK 20140750.2, and **F** specimen CUMZ 6273, from Xieng Khuang. **G** *Haploptychius fischeri*, lectotype MNHN-IM 200030873.

Figs 1 , 2A , 3A–C , 7A, B , 8A–D , 9A–F , 10G ; Table 1

Streptaxis pellucens Pfeiffer 1863 [1862]: 273, pl. 36, fig. 6. Type locality: Lao Mountain, Camboja [Cambodia]. Martens 1867: 85. Pfeiffer 1868: 441. Pfeiffer 1871: 29, pl. 115, figs 11, 12. Morlet 1883: 105, pl. 4, fig. 2, 2a. Tryon 1885: 71, 72, pl. 14, figs 98–100. Gude 1903: 212.
Haploptychius pellucens —Kobelt 1906: 132, 133, pl. 61, figs 17–20. Richardson 1988: 217, 218. Schileyko 2011: 24, 25.

##### Material examined.

This species was described from the H. Cuming collection. An illustration of the shell and one set of measurements were given in the original description. Three specimens from the Cuming collection at NHM have Pfeiffer’s handwritten label stating the species name and locality. In order to stabilise the name, an identical specimen matching with the illustration and measurements given in the original description is designated here as lectotype NHMUK 20160249.1 (Fig. 3A; H = 11.7, W = 11.2). The other two remaining shells from the same lot then became paralectotypes NHMUK 20160249.2 (2 shells; Fig. 3B; H = 11.1, W = 10.6 and H = 13.1, W = 13.4).

**Table 1.** Shell measurements for populations of the three *Haploptychius*, four *Perrottetia*, and one *Indoartemon* species collected.

Specie and locality and CUMZ nos	No. of specimens	Rangs, mean ± S.D. in mm of:				Number of whorls
		Shell height (H)	Shell width (W)	H/W ration	Shell angle	
*Haploptychius pellucens* (Pfeiffer, 1863)						
Lectotype and paralectotypes	3	11.1−13.0 11.9±1.02	10.6−13.3 11.7±1.45	0.9−1.0 1.0±0.01	31.7−37.1 34.9±2.77	6−6½
1. Ban Namone, Xayabouly (about 40 Km. from Ngeun, Lao-Thai border to Xayabouly district): (6264, 6265)	12	10.1−12.10 11.0±0.55	9.6−12.0 10.6±0.63	0.9−1.18 1.0±0.09	33.6−53.02 44.2±5.26	6−6½
2. Tam Phatok, Luang Phrabang: (6267)	7	9.5−10.7 9.8±0.43	9.0−10.7 9.8±0.75	0.9−1.1 1.0±0.11	45.8−56.9 50.9±3.75	6−6½
3. Ngoi, Luang Phrabang: (6268)	7	9.7−12.4 11.1±0.90	10.4−11.8 10.9±0.48	0.9−1.1 1.0±0.01	48.8−54.1 51.2±1.91	6½
4. Nam Ork Roo, Nathong, Namor, Oudomxay: (6269, 6270)	35	9.5−11.5 10.2±0.53	9.3−12.0 10.8±0.61	0.8−1.1 0.9±0.08	42.5−54.1 48.9±3.19	6½
5. Ban Oudom, Pak Beng, Oudomxay: (6271)	15	10.6−13.1 12.0±0.77	9.5−12.8 11.2±0.76	0.8−1.3 1.0±0.12	37.6−58.3 47.9±5.21	6½−7
7. Tam Mungkorn, Khamkeurt, Bolikhamxay: (6266)	4	8.8−9.6 9.3±0.30	8.0−9.1 8.7±0.50	1.0−1.1 1.0±0.03	46.7−50.3 48.2±1.56	6½−7
*Haploptychius porectus* (Pfeiffer, 1863)						
8. Ban Nong Tang, Phoukood, Xieng Khuang: (6273, 6274)	19	6.2−8.1 7.4±0.50	6.3−8.4 7.4±0.52	0.8−1.2 0.9±0.11	41.1−59.6 49.6±4.58	6½
9. Tam Pew, Kham, Xieng Khuang: (6275)	4	6.5−7.2 7.0±0.34	7.3−8.5 7.7±0.59	0.8−0.9 0.9±0.06	44.1−47.1 45.0±1.52	6½
*Haploptychius blaisei* (Dautzenberg & Fischer, 1905)						
10. Tam Phatok, Ngoi, Luang Phrabang: (6276, 6277)	16	5.4−6.7 6.2±0.35	9.1−10.5 9.8±0.36	0.5−0.7 0.6±0.05	53.7−75.3 67.1±5.9	6½
*Perrottetia aquilonaria* Siriboon & Panha, 2013						
12. Ban Namone, Xayabouly (about 40 Km. from Ngeun, Lao-Thai border to Xayabouly District): (6278, 6279)	3	4.1−4.5 4.4±0.19	5.4−6.4 6.0±0.55	0.7−0.9 0.7±0.09	48.4−59.1 54.2±5.43	5½−6
*Perrottetia unidentata* sp. n.						
13. Ban Nawit, Viengxay: (6281, 6282, 6283)	8	4.0−5.8 5.0±0.05	8.9−9.7 9.3±0.25	0.4−0.6 0.5±0.06	67.0−88.9 76.8±6.74	6−6½
14. Tam Than Kaisone Phomvihan, Viengxay: (6284, 6285)	5	5.0−6.5 5.9±0.77	7.4−8.2 7.7±0.36	0.6−0.8 0.7±0.11	54.5−60.7 57.1±2.51	6
*Perrottetia magnadenta* sp. n.						
15. Km 70, Tha Khek, Yommalat: (6286, 6287)	36	6.0−7.6 6.7±0.36	7.2−8.8 7.8±0.42	0.7−0.9 0.8±0.06	47.4−59.9 54.5±3.34	6
*Indoartemon diodonta* sp. n.						
16. Tam Xang, Tha Khek, Khammouan: (6289, 6290)	49	6.8−8.0 7.4±0.33	6.9−8.6 7.7±0.37	0.8−1.0 0.9±0.06	42.1−58.1 51.8±3.03	6½−7
17. Tam Nang Ann, Tha Khek, Khammouan: (6291)	7	7.9−8.9 8.6±0.33	7.2−8.3 7.8±0.34	1.0−0.1 1.1±0.03	46.4−52.4 49.7±2.07	6½−7
18. Tam Xieng Lieb, Tha Khek, Khammouan: (6292)	15	6.8−7.8 7.3±0.27	6.4−7.8 7.3±0.46	0.8−1.1 1.0±0.09	41.3−61.0 51.8±5.04	7

Cambodia: NHMUK MacAndrew coll. (4 shells). Ban Namone, Xayabouly, Laos (about 40 Km. from Ngeun District, Lao-Thai border to Xayabouly District): CUMZ 6264 (Fig. 3C; 8 shells), 6265 (4 specimens in ethanol). Tam Phatok, Ngoi, Luang Phrabang, Laos: CUMZ 6267 (7 shells). Ngoi, Luang Phrabang, Laos: CUMZ 6268 (7 shells). Nam Ork Roo, Nathong, Namor, Oudomxay, Laos: CUMZ 6269 (29 shells), 2670 (6 specimens in ethanol; Figs 3A, B, 8A, 9A–F, 10G). Ban Oudom, Pak Beng, Oudomxay, Laos: CUMZ 6271 (15 shells). Tam Kao Rao, Vieng Phoukha, Luang Namtha, Laos: CUMZ 2672 (2 shells). Tam Mung Korn, Khamkeurt, Bolikhamxay, Laos: CUMZ 6266 (4 shells).

##### Description.

**Shell.** Shell oblique-ovate, white and translucent. Whorls 6½, spire conical with distinct suture. Shell surface glossy with thin transverse ridges which diminish below periphery. Embryonic shell about 2½ whorls, with smooth surface; following whorls regularly coiled. Penultimate whorl and last whorl rounded, axially deflected. Aperture subcircular; peristome thin, little expanded and reflected. Apertural dentition with one more or less strong parietal lamella. Umbilicus open and deep (Fig. 3A–C).

**Radula.** Each row consists of 77–85 teeth with formula (38-42)-1-(38-42). Central tooth very small, triangular, with a pointed cusp. Lateral and marginal teeth undifferentiated, lanceolate, unicuspid. Latero-marginal teeth gradually reduce in size, with outermost teeth much smaller and shorter than inner teeth (Fig. 10G).

**Genital organs.** Atrium (at) short. Proximal penis (p) stout about one-thirds of penis length; distal penis slender. Penial sheath (ps) thin, extending about half of penis length; penial sheath retractor muscle (psr) very thin, originating at atrium and inserted apically at penial sheath (Fig. 6A). Vas deferens (vd) passes through about one-third of penial sheath length before entering into penis apically. Penial retractor muscle (pr) very thick and connected at penis apically (Fig. 7A, B).

Internal wall of atrium generally corrugated with numerous atrial pores (Fig. 9A). Penial wall densely covered in light brown penial hooks, about 6 hooks/200 μm^2^. Hooks located on low conical penial papillae, separated by thin reticulated folds. Penial hooks small (< 0.1 mm in length), long, slender, slightly expanded at base, tips pointed and curved towards genital orifice (Fig. 9B–E).

Vagina (v) short, about one-third of penis length. Gametolytic duct (gd) long tube extending to albumin gland; gametolytic sac (gs) ovate. Free oviduct (fo) proximally large with almost equal diameter to vagina, becoming narrower distally. Oviduct (ov) enlarged and folded; prostate gland inconspicuous and bound to oviduct. Talon (ta) small, short and club-shaped. Hermaphroditic duct (hd) bearing long and thin seminal vesicle. Seminal vesicle (sv) about three times longer than the length from talon to branching point of seminal vesicle (Fig. 7A).

Vaginal wall with series of transverse and undulated parallel vaginal folds; vaginal hooks absent (Fig. 9F).

##### Distribution.

This species is known from several limestone areas from central to northern part of Laos. The animals can be found at altitudes from 150-300 meters above mean sea level.

##### Remarks.

This species can be distinguished from *Haploptychius porrectus* by having a larger shell, more elevated spire elevated and less oblique aperture. The vas deferens passes through a shorter part of the penial sheath length, and the vagina wall has undulated transverse ridges rather than papillae. *Haploptychius pellucens* can be distinguished from *Haploptychius costulatus* (Möllendorff, 1881) from China by having a larger and thinner shell, narrower umbilicus andhaving the left periphery of the penultimate whorl extending beyond the diameter of the last whorl. *Haploptychius fischeri* differs from this species by having a larger, more depressed and thicker shell, with a more obtuse spire, and subquadrangular aperture (Fig. 3G).

All live adult specimens were dissected and the genitalia have been examined, and three different types of genitalia are observed. There are six fully adult specimens collected from Nam Ork Roo, Oudomxay with ‘normal’ genitalia (Fig. 8A). Two specimens from Ban Namone, Xayabouly have no male genital organs (penis, retractor muscle, vas deferens and prostate gland), while female genital organs are well developed and fully function (Fig. 8D). This is apparently the first report of aphallic animals in Streptaxidae. The other two specimens from Ban Namone, have a ‘normal’ penis, but have an enlarged and curved “vaginal caecum (vc)” near the penis and atrium junction (Fig. 8B, C). This too is an unusual or unique structure in Streptaxidae. Nevertheless, all these animals appear conspecific based on their shells and the causes of this variation are unknown.

#### Haploptychius porrectus

Taxon classificationAnimaliaPulmonataStreptaxidae

(Pfeiffer, 1863)

Figs 1 , 2B , 3D–F , 7C, D , 9G–M , 10H ; Table 1

Streptaxis porrecta Pfeiffer 1863 [1862]: 273. Type locality: Lao Mountains, Camboja [Cambodia]. Martens 1867: 85. Pfeiffer 1868: 442. Tryon 1885: 74. Fischer 1891: 18. Gude 1903: 217. Gude 1903: 275, 322, 325, pl. 12, figs 20–22.
Haploptychius porrectus —Kobelt 1906: 133, pl. 61, figs 24-26. Richardson 1988: 219.

##### Material examined.

This species was described from the H. Cuming collection. The number of specimens was not indicated, but only one set of measurements was given in the original description. The NHM collection contains two specimens from the Cuming collection that has Pfeiffer’s handwritten label stating the species name and collection locality. In order to stabilize the name, a specimen matching with the measurements given in the original description is designated here as lectotype NHMUK 20140750.1 (Fig. 3D; H = 8.0, W = 9.0). The other specimen from the same lot becomes a paralectotype NHMUK 20140750.2 (1 shell; Fig. 3E; H = 8.0, W = 8.2).

Laos: NHMUK 1906.1.1.770 (4 shells), NHMUK MacAndrew coll. (2 shells). Ban Nong Tang, Phoukood, Xieng Khuang, Laos: CUMZ 6273 (18 shells; Fig. 3F), 6274 (1 specimen in ethanol; Figs 2B, 7C, D). Tam Pew, Kham, Xieng Khuang, Laos: CUMZ 6275 (4 specimens in ethanol; Figs 9G–M, 10H).

##### Description.

**Shell.** Shell oblique-heliciform, white and translucent. Whorls 6½, spire conical, suture distinct. Shell surface glossy, with transverse ridges that diminish below the periphery. Embryonic shell smooth with 2½ whorls; following whorls regularly coiled. Penultimate whorl rounded; last whorls rounded and axially deflected. Aperture subcircular; peristome thickened and reflected. Aperture dentition with one parietal lamella. Umbilicus open and deep (Fig. 3D–F).

**Radula.** Each row consists of 46–58 teeth with formula (23-29)-1-(23-29). Central tooth very small and triangular, with a pointed cusp. Lateral and marginal teeth undifferentiated, lanceolate, unicuspid. Latero-marginal teeth gradually reduce in size, with outermost teeth much smaller and shorter than inner teeth (Fig. 10H).

**Genital organs.** Atrium (at) short. Proximal penis (p) stout about one-fifth of penis length; distal penis slender. Penial sheath (ps) thin, extending about two thirds of penis length; penial sheath retractor muscle (psr) very thin, originating at atrium and inserting distally on penial sheath (Fig. 7C). Vas deferens (vd) passes through about a quarter of the penial sheath length before entering into penis apically (Fig. 7D). Penial retractor muscle (pr) thick, short and connected with penis apically.

Internal wall of atrium generally smooth (Fig. 9G). Proximal penial wall densely covered with brownish penial hooks, about 10 hooks/200 μm^2^. Hooks located on low conical penial papillae, separated by very thin reticulated folds. Proximal penial hooks small and short (< 0.04 mm in length), slightly expanded at base, tip sharp and directed towards genital orifice (Fig. 9H, I). Distal penial wall less densely scattered with brownish penial hooks, about 4 hooks/200 μm^2^; penial papillae absent. Distal hooks very large, long and slender (< 0.5 mm in length), expanded at base, tip obtuse and directed towards genital orifice (Fig. 9J, K).

Vagina (v) short, about half of penis length. Gametolytic duct (gd) long tube extending as far as albumin gland; gametolytic sac (gs) small. Free oviduct (fo) short with almost the same diameter as vagina. Oviduct (ov) enlarged and folded; prostate gland inconspicuous and bound to oviduct. Talon (ta) small and club shape. Hermaphroditic duct (hd) bearing very long and enlarged seminal vesicle (sv) about ten times longer than the length from talon to branching point of seminal vesicle (Fig. 7C).

Vaginal wall generally corrugated with irregular vaginal papillae (Fig. 9L, M). Vaginal hooks absent.

##### Distribution.

This species is known from the limestone outcrops in northeastern and central parts of Laos. The animals can be found at altitudes from 150-300 meters above mean sea level.

##### Remarks.

This species can be distinguished from *Haploptychius dorri* (Dautzenberg, 1894) and *Haploptychius blaisei* (Dautzenberg & Fischer, 1905) in having a less depressed shell and less deviated last whorl. In addition, *Haploptychius blaisei* possesses a solid shell with an angular penultimate whorl, and *Haploptychius dorri* has a smaller and smooth shell with an angular penultimate whorl. *Haploptychius anceyi* (Mabille, 1887) is similar to *Haploptychius porrectus*, however it differs in its smaller shell, circular aperture, and nearly smooth shell surface.

#### Haploptychius fischeri

Taxon classificationAnimaliaPulmonataStreptaxidae

(Morlet, 1887)

Fig. 3G

Streptaxis fischeri Morlet 1887 [1886]: 259, 274, pl. 12, fig. 1, 1a. Type locality: Baie ďHalong et Montagne de ľÉléphant [Elephant Mountain of Halong Bay, Quang Ninh Province, Vietnam]. Gude 1903: 212.
Haploptychius fischeri —Kobelt 1906: 136, pl. 61, fig. 21, Richardson 1988: 215. Schileyko 2011: 25.

##### Material examined.

The species was described based on material from Jourdy’s collection and an illustration was included in the original description (Morlet 1887: 259, pl. 12, fig. 1, 1a). There is a single specimen from L. Morlet in the MNHN collections with an original label stating “Type”. In order to stabilize the name, a shell that matched well with the illustration and measurements given in the original description is designated here as lectotype MNHN-IM 200030873 (Fig. 3G).

##### Remark.

*Haploptychius fischeri* is currently known only from the north of Vietnam (Schileyko 2011, Do and Do 2015). The type specimen was examined. Shell thickened, oblique-heliciform with depressed spire. Shell surface with strong radial ridges; penultimate whorl rounded; last whorl axially deflected. Aperture subquadrangular, parietal lamella strong and parietal callus thickened. Peristome wide; lip thickened and reflected. Umbilicus narrowly open.

Compared with *Haploptychius pellucens* and *Haploptychius porrectus*, this species differs in its larger and thicker shell, depressed spire, prominent transverse ridges, subquadrangular aperture, thicker parietal lamella, and narrower umbilicus.

#### Haploptychius blaisei

Taxon classificationAnimaliaPulmonataStreptaxidae

(Dautzenberg & Fischer, 1905)

**Figure 4.**
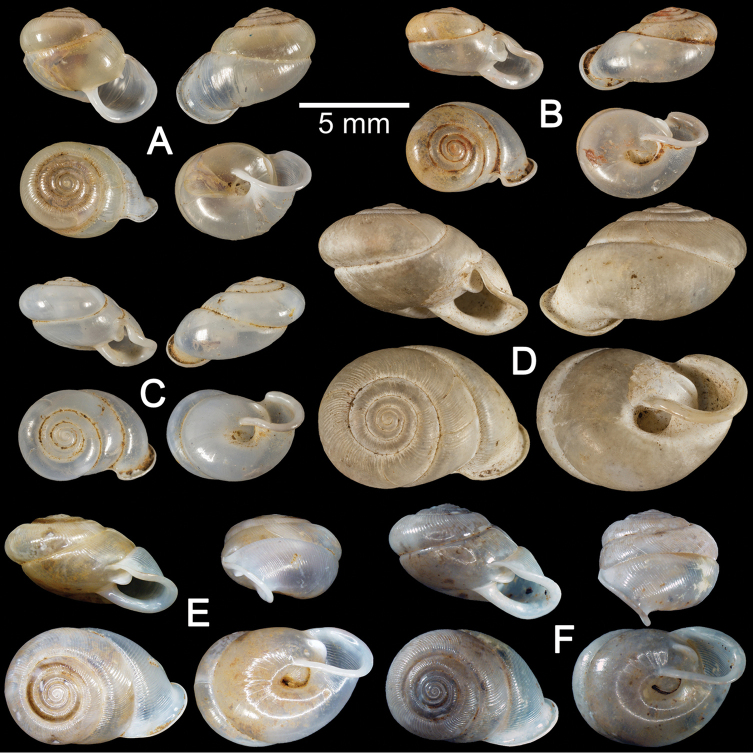
Shells of *Haploptychius* spp. **A** *Haploptychius anceyi* lectotype MNHN-IM 200030868 **B** *Haploptychius diespiter* syntype MNHN-IM 200030870 **C** *Haploptychius dorri* syntype MNHN-IM 200030869 **D–F** *Haploptychius blaisei* **D** holotype MNHN-IM 200030866 and **E, F** specimens from Luang Phrabang CUMZ 6257.

Figs 1 , 4D–F ; Table 1

Streptaxis blaisei Dautzenberg and Fischer 1905: 86, 87, pl. 3, figs 1–4. Type locality: Ile Krieu, Tonkin [Krieu Island, Ha Long, Quang Ninh Province, Vietnam].
Haploptychius blaisei —Kobelt 1906: 173, pl. 66, figs 4–7. Richardson 1988: 212.

##### Material examined.

The original description was based on single specimen since stated “un seul examplaire” (a single example). The specimen of M. Blaise in the MNHN collections is considered as holotype MNHN-IM 200030866 (Fig. 4D).

Phu Ly, Dongson, Ha Nam, Vietnam: NHMUK Vermeulen coll. (4 shells). Tam Phatok, Ngoi, Luang Phrabang, Laos: CUMZ 6276 (1 shell; Fig. 4E), 6257 (15 shells; Fig. 4F).

##### Remarks.

Shell oblique-heliciform, white and translucent. Whorls 6½; spire depressed to slightly convex, with distinct suture. Shell surface glossy, with thin transverse ridges that diminish below periphery and around umbilicus. Embryonic shell large, about 2½ whorls, with a smooth surface; following whorls regularly expanded. Penultimate whorl rounded; last whorl axially deflected. Aperture semi-ovate; peristome discontinuous, parietal callus thin; lip thickened and slightly expanded. Apertural dentition with one strong parietal lamella. Umbilicus widely open and shallow.

*Haploptychius blaisei* is superficially similar to *Haploptychius diespiter* (Mabille, 1887) and *Haploptychius dorri* from north Vietnam, but it has a larger shell, more depressed spire, rounded penultimate whorl, a wide and deep umbilicus, and thin transverse ridges on the upper periphery. For comparison, *Haploptychius diespiter* (Fig. 4B) has the last whorl less deviated from the vertical axis, and *Haploptychius dorri* (Fig. 4C) has a more depressed suture.

#### Genus Perrottetia

Taxon classificationAnimaliaPulmonataStreptaxidae

Kobelt, 1905

Odontartemon (Perrottetia) Kobelt 1905: 91. Kobelt 1906: 108. Thiele 1931: 730. Forcart 1946: 215.
Oophana (Perrottetia) —Benthem Jutting 1954: 95.
Perrottetia —Zilch 1960: 562, 563, Richardson 1988: 237. Schileyko 2000: 777, 778. Siriboon et al. 2013: 44, 45.

##### Type species.

*Helix peroteti* Petit, 1841, by subsequent designation of Forcart (1946: 215).

##### Remarks.

The genus *Perrottetia* differs from all other Southeast Asian streptaxid genera in having two longitudinal furrows outside the aperture. Apertural dentition usually comprises one or two parietal lamellae, plus, palatal, basal and columellar lamellae. Genitalia with long penis, penial hooks present, and vaginal hooks sometimes present (Zilch 1960, Schileyko 2000, Siriboon et al. 2013).

Currently, 29 *Perrottetia* species are recognized, from India and Sri Lanka to Indochina and southern China (Kobelt 1906, Richardson 1988, Schileyko 2011, Siriboon et al. 2013). Two species have been reported from Laos, *Perrottetia dugasti* (Morlet, 1892) and *Perrottetia daedaleus* (Bavay & Dautzenberg, 1908) (see Schileyko 2011).

#### Perrottetia dugasti

Taxon classificationAnimaliaPulmonataStreptaxidae

(Morlet, 1892)

Fig. 5A

Streptaxis dugasti Morlet 1892: 82. Morlet 1893[1892]: 315, 316, pl. 7, fig. 5, 5a, 5b. Type locality: Laï-Chau, sur les bords de la Riviére Noire, Tonkin [on the banks of the Black River, Lai Chau Province, Vietnam]. Gude 1903: 255.
Perrottetia dugasti —Kobelt 1906: 123, 124, pl. 61, fig. 13. Richardson 1988: 239. Schileyko 2011: 23.

##### Material examined.

The species was described based on material from L. Dugast collection but no illustration was given. Morlet (1893: 315, 316, pl. 7, fig. 5, 5a, 5b) subsequently published the description and illustrated a single specimen. There is a specimen of L. Morlet in the MNHN collections with an original label stating “Type”. In order to stabilise the name, the shell that closely matched with the measurements given in the original description and illustration in Morlet (1893: pl. 7, fig. 5, 5a, 5b) is here designated as lectotype MNHN-IM 200030867 (Fig. 4A).

##### Remarks.

Shell sub-oblique heliciform with depressed spire and 6 whorls. Shell surface smooth, glossy and with a distinct suture. Embryonic shell smooth, following whorl regularly expanded. Last whorl rounded, axially deflected, with longitudinal furrows present. Aperture narrow; peristome discontinuous, thick and expanded, and short sinulus present. Aperture dentition consisting of two parietal lamellae (lower one large; upper one small and close to sinulus), one palatal lamella, one basal lamella and one bifid columellar lamella.

**Figure 5.**
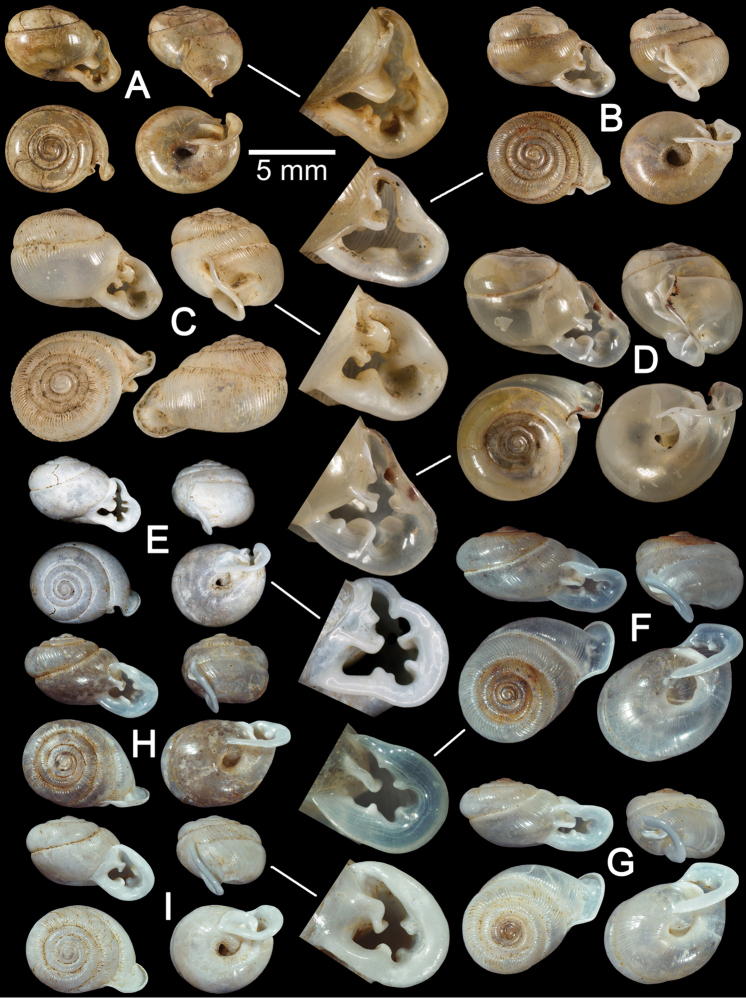
Shells of *Perrottetia* spp. **A** *Perrottetia dugasti* lectotype MNHN-IM 200030867 **B** *Perrottetia mabillei* syntype MNHN-IM 200030874 **C** Perrottetia daedaleus var. major syntype MNHN-IM 200030871 **D** *Perrottetia messageri* syntype MNHN-IM 200030875 **E** *Perrottetia aquilonaria* specimen CUMZ 6278 from Xayabouly with apertural dentition **F, G** *Perrottetia unidentata* sp. n. **F** holotype CUMZ 6281 with apertural dentition and **G** paratype CUMZ 6282 **H, I** *Perrottetia unidentata* sp. n. specimens from Tam Than Kaisone **H** specimen with upper palatal CUMZ 6284 and **I** specimen without upper palatal CUMZ 6285.

Compared with *Perrottetia messageri* (Bavay & Dautzenberg, 1908), this species differs in having a strong lower parietal lamella, a bifid columellar lamella, and the left periphery of penultimate whorl not extended beyond the diameter of the last whorl. In contrast, *Perrottetia messageri* has a strong columellar lamella, a supracolumellar lamella is present, and the left periphery of the penultimate whorl extended beyond the diameter of the last whorl (Fig. 5D).

#### Perrottetia daedaleus

Taxon classificationAnimaliaPulmonataStreptaxidae

(Bavay & Dautzenberg, 1908)

Fig. 5C

Streptaxis daedaleus Bavay and Dautzenberg 1908: 230. Type locality: Pac-Kha [Pa Kha, Son La Province, Vietnam]. Bavay and Dautzenberg 1909: 164, 165, pl. 4, figs 1–4.
Streptaxis daedaleus var. major Bavay and Dautzenberg 1908: 231. Type locality: Pac-Kha [Pa Kha, Son La Province, Vietnam]. Bavay and Dautzenberg 1909: 165.
Oophana daedaleus —Richardson 1988: 234. Schileyko 2011: 23.
Oophana daedaleus major —Richardson 1988: 234.

##### Material examined.

Syntype of Streptaxis daedaleus var. major MNHN-IM 200030871 (Fig. 5B). Tonkin: NHMUK 1909.6.9.118-9 (2 shells). Pac-Kha, Tonkin: NHMUK 1909.7.9.15-6 (2 shells), NHMUK Preston coll. date 7.4.09 (2 shells), Rolle coll. date 27.11.09 (3 shells). Long Ping, Tonkin: NHMUK Rolle coll. date 27.1.09 (2 shells).

##### Remarks.

Shell suboblique-heliciform with a convex spire and 6 whorls. Shell surface with strong transverse ridges running continuously to umbilicus. Embryonic shell with thin transverse ridges and following whorl regularly expanded. Last whorl rounded, axially deflected, longitudinal furrows present. Aperture triangular; peristome discontinuous, thickened, broadly expanded and sinulus absent. Apertural dentition with two parietal lamellae (lower one small; upper one large and close to sinulus), one angular lamella, one palatal lamella (located far inside aperture) and one columellar lamella.

This species is superficially similar to *Perrottetia mabillei* (Bavay & Dautzenberg, 1903) in having strong transverse ridge over the entire shell, but *Perrottetia daedaleus* has a large upper parietal lamella, a palatal lamella located inside the aperture, and strong columellar lamellae, while *Perrottetia mabillei* (Fig. 5B) has a large lower parietal lamella and bifid columellar lamellae.

#### Perrottetia aquilonaria

Taxon classificationAnimaliaPulmonataStreptaxidae

Siriboon & Panha, 2013

Figs 1 , 5E

Perrottetia aquilonaria Siriboon et al. 2013: 50–52, figs 3D–H, 4D–F: Type locality: Wat Tam Pha Plong, Chiangdao, Chiangmai, Thailand.

##### Material examined.

Holotype CUMZ 5003, paratypes CUMZ 5004 (4 shells). Ban Namone, Xayabouly, Laos: CUMZ 6278 (2 shells; Fig. 5E), CUMZ 6279 (1 specimen in ethanol). Ban Bo Khoun, Boun Neua, Phongsaly, Laos: CUMZ 6280 (1 shell).

##### Remarks.

*Perrottetia aquilonaria* was described from several localities in the northern part of Thailand with a complete information on shell, radula and genitalia. The specimens collected from limestone outcrops in Borkeo and Phongsaly of Laos have both shells and genitalia that match very well with this species. Laos specimens seem to differ only in the slightly smaller shell, therefore we treated them as the same species.

*Perrottetia aquilonaria* can be distinguished from *Perrottetia dugasti* and *Perrottetia messageri* from Vietnam by having a depressed spire, shouldered last whorl, thin parietal callus and upper-parietal lamella separated at a right angle. In contrast, *Perrottetia dugasti* has a rounded last whorl and a small upper-parietal lamella located deeper inside the aperture, and *Perrottetia messageri* has paralleled parietal lamellae, a small supercolumellar lamella is present, and the left side of the penultimate whorl extended beyond the diameter of the last whorl (Fig. 5A, D).

#### Perrottetia unidentata

Taxon classificationAnimaliaPulmonataStreptaxidae

Inkhavilay & Panha sp. n.

http://zoobank.org/B47C107D-B7A5-4D70-8640-47F10AE13AC7

Figs 1 , 5F–I , 7E, F , 10A–F, I ; Table 1

##### Type material.

Holotype CUMZ 6281 (Fig. 5F). Measurement: shell height 5.3 mm, shell width 9.7 mm and 6½ whorls. Paratypes CUMZ 6282 (4 shells; Fig. 5G), CUMZ 6283 (1 specimen in ethanol; Figs 7E, F, 9A–F, I), NHMUK 20160250 (2 shells).

**Figure 6.**
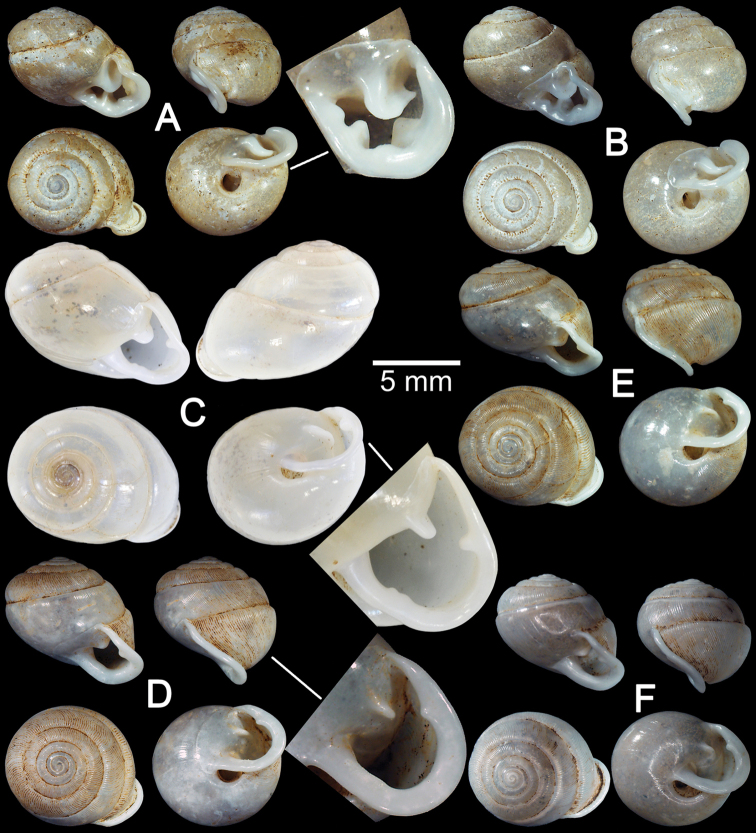
Shells of *Perrottetia* and *Indoartemon* spp. **A, B** *Perrottetia megadentata* sp. n. **A** holotype CUMZ 6286 with apertural dentition, and **B** paratype CUMZ 6287. **C** *Indoartemon tridens* holotype SMF 108507/1 with apertural dentition **D–F** *Indoartemon diodonta* sp. n. **D** holotype CUMZ 6289 with apertural dentition **E** paratypes CUMZ 6290, and **F** specimen from Tam Nang Ann, Tha Khek, Khammouan CUMZ 2691.

**Figure 7.**
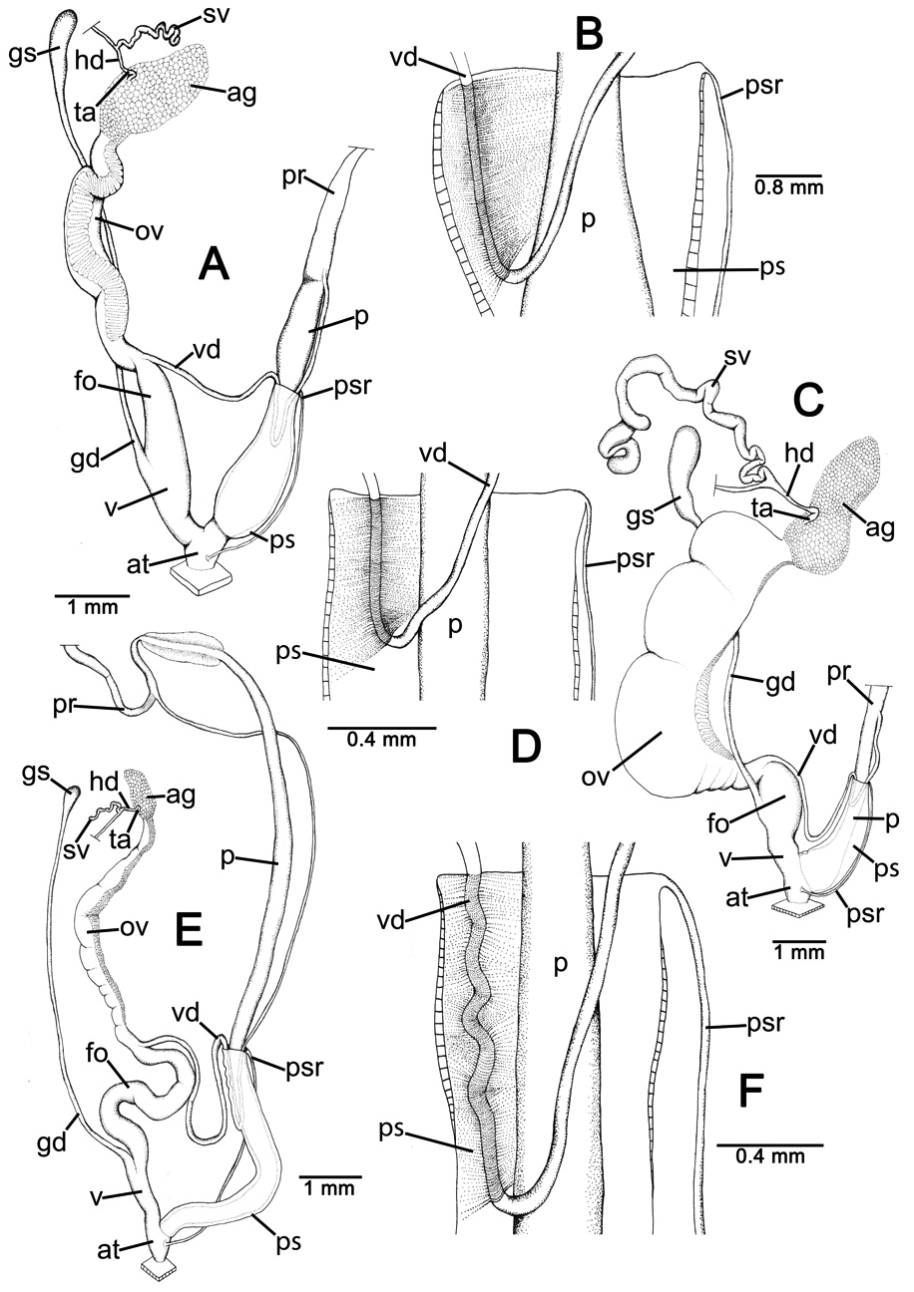
Genitalia of *Haploptychius* and *Perrottetia* species. **A, B** *Haploptychius pellucens* CUMZ 2670 **A** reproductive system, and **B** insertion of vas deferens into penial sheath **C, D** *Haploptychius porrectus* CUMZ 6274 **C** reproductive system, and **D** insertion of vas deferens into penial sheath **E, F** *Perrottetia unidentata* sp. n. CUMZ 6283 **E** reproductive system, and **F** insertion of vas deferens into penial sheath.

**Figure 8.**
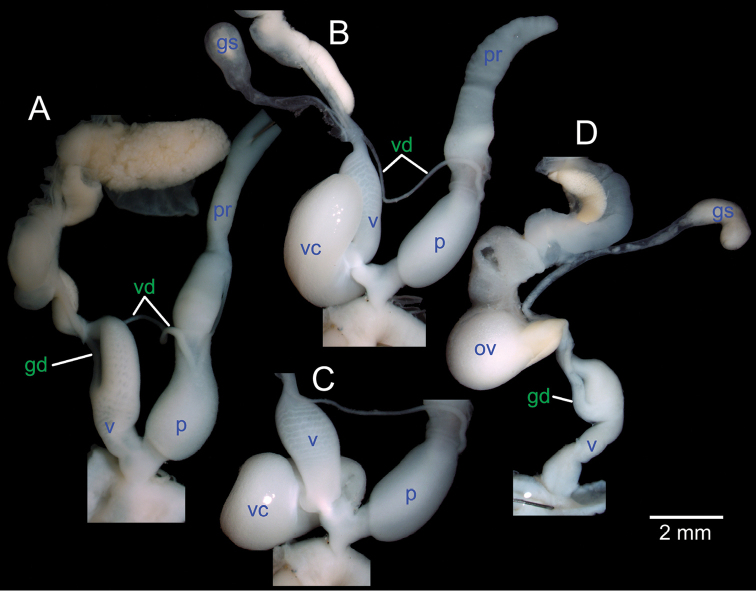
Genitalia of *Haploptychius pellucens* **A** completed reproductive system CUMZ 2670 **B, C** completed reproductive system with “vaginal caecum” CUMZ 6265, and **D** aphallic reproductive system CUMZ 6265.

**Figure 9.**
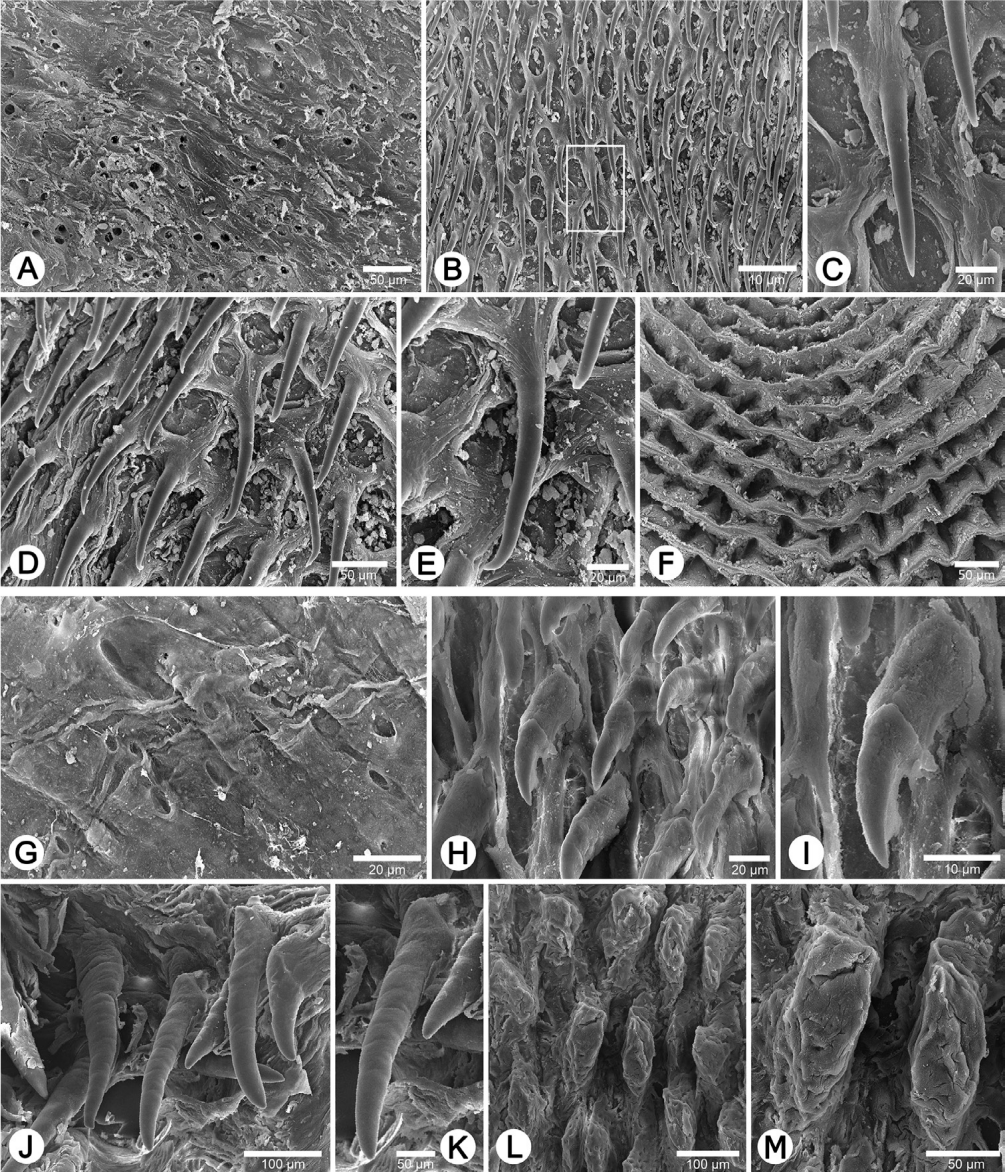
Internal sculpture of genitalia of *Haploptychius* spp. **A–F** *Haploptychius pellucens*, CUMZ 2670 **A** details of atrium surface **B** arrangement of penial hooks **C** top view of penial hook (from white square in **B**) **D** arrangement of penial hooks **E** lateral view of penial hook, and **F** arrangement of undulated parallel vaginal folds. **G–M** *Haploptychius porrectus* specimen CUMZ 6275 **G** details of atrium surface **H** arrangement of penial hooks in distal area **I** lateral view of penial hook in distal area **J** arrangement of penial hooks in proximal area **K** lateral view of penial hook in proximal area **L** arrangement of papillae and vaginal folds, and **M** arrangement of vaginal folds.

##### Other material examined.

Tam Than Kaisone, Viengxay, Houaphanh, Laos: CUMZ 6284 (5 shell; Fig. 5I), CUMZ 6285 (2 shells; Fig. 5H).

##### Type locality.

The limestone outcrop at Ban Nawit, Viengxay, Houaphanh, Laos (20°22'37.3"N, 104°16'43.2"E) about 700 meters above mean sea level.

##### Diagnosis.

This new species differs from *Perrottetia daedaleus*, *Perrotettia aquilonaria*, *Perrottetia dugasti* and *Perrottetia messageri* from Vietnam in having an oblique shell, a single parietal lamella, widely expanded lip, the last whorl strongly axially deflected, the left side of penultimate whorl well extended beyond the diameter of last whorl, and the distal end of penis with a wing-like structure. The other four species have two parietal lamellae, the last whorl little axially deflected and the left side of penultimate whorl not extended beyond the diameter of the last whorl. For further comparison, *Perrottetia daedaleus* has an elevated spire, transverse ridges over the entire shell and a basal lamella located deep inside aperture (Fig. 5C); *Perrotettia aquilonaria* has a smaller shell, elevated spire, bifid columellar lamella, and genitalia with atrial pores and vaginal hooks absent (Fig. 5E); *Perrottetia dugasti* and *Perrottetia messageri* have a smooth shell surface, a bifid collumella lamella and a supracolumellar lamella (Fig. 5A, D). *Perrottetia gudei* from north Vietnam differs from the new species in having an elevated spire, in being less deviated from the vertical axis, and in having thin transverse ridges (see Siriboon et al. 2013).

##### Description.

**Shell.** Shell oblique-heliciform, semi-transparent; whorls 6½, spire weakly convex with distinct suture. Shell surface glossy with strong transverse ridges on upper shell surface. Embryonic shell large, about 2½ whorls, with a smooth surface; following whorls regularly coiled. Shell periphery shouldered; last whorl axially deflected; two deep longitudinal furrows present. Aperture semi-ovate; peristome discontinuous; parietal callus thin; lip thickened, broadly expanded and slightly reflected. Apertural dentition with one large, strong and sinuous parietal lamella, one small upper palatal lamella, one palatal lamella, one large basal lamella, one strong columellar lamella, and one small supracolumellar lamella. Umbilicus widely open and shallow (Fig. 5F–I).

**Figure 10.**
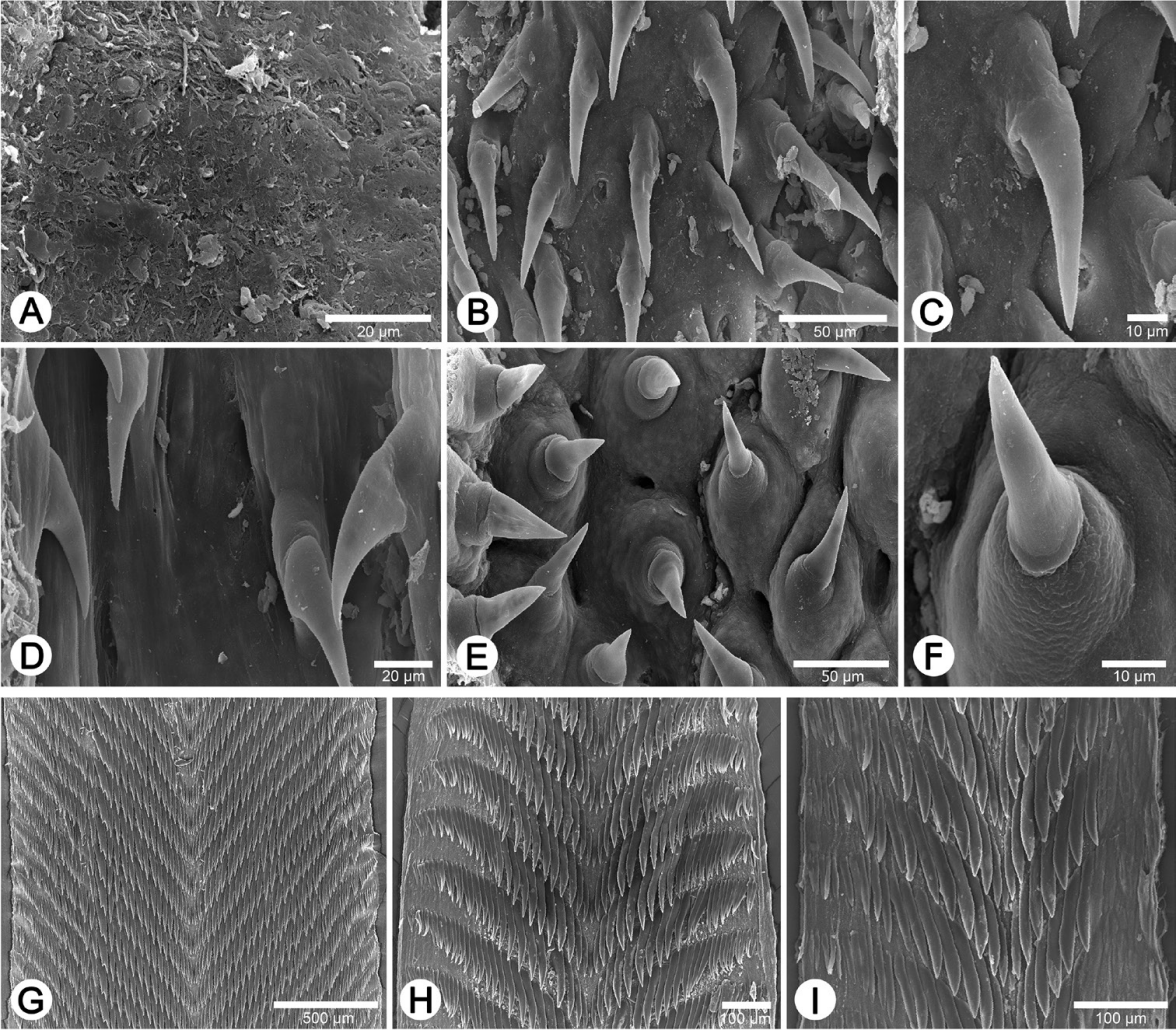
Internal sculpture of genitalia of **A–F** *Perrottetia unidentata* sp. n. paratype CUMZ 6283 **A** details of atrium surface **B** arrangement of penial hooks **C** top view of penial hook **D** lateral view of penial hooks **E** arrangement of vaginal hooks, and **F** top view of vaginal hook. Radula morphology of **G** *Haploptychius pellucens* specimen CUMZ 2670 **H** *Haploptychius porrectus* specimen CUMZ 6275, and **I** *Perrottetia unidentata* sp. n. paratype CUMZ 6283.

**Radula.** Each row consists of 26–38 teeth with formula (13-19)-1-(13-19). Central tooth small and triangular, with pointed cusp. Lateral and marginal teeth undifferentiated, lanceolate, unicuspid. Latero-marginal teeth gradually reduce in size, with outermost teeth much smaller and shorter than inner teeth (Fig. 10I).

**Genital organs.** Atrium (at) short. Proximal penis (p) long and slender; distal part near retractor muscle with an expanded wing-like structure (a flat blade on either side of the penis, each about one-tenth of penis length). Penial sheath (ps) thin and extending about one-third of penis length; penial sheath retractor muscle (psr) very thin, originating at atrium and inserting distally on penial sheath (Fig. 7E). Vas deferens (vd) passes through about one-third of penial sheath length before entering into penis apically (Fig. 7F). Penial retractor muscle (pr) thin and long, inserted at penis and vas deferens junction.

Internal wall of atrium generally smooth (Fig. 10A). Penial wall densely covered with light brown penial hooks, about 20 hooks/200 μm^2^; hooks located on low elliptical penial papillae. Penial hooks small (< 0.1 mm in length), slender, expanded at base, tips pointed and curved towards genital orifice (Fig. 10B–D).

Vagina (v) short, about one-tenth of penis length. Gametolytic duct (gd) a long tube extending as far as albumin gland; gametolytic sac (gs) ovate. Free oviduct (fo) long and cylindrical with equivalent diameter to vagina, tapering distally. Oviduct (ov) enlarged and folded; prostate gland inconspicuous and bound to oviduct. Talon (ta) very small, short and club shape. Hermaphroditic duct (hd) bearing very short and thin seminal vesicle (sv) about one and half times longer than the length from talon to branching point of seminal vesicle (Fig. 7E).

Vaginal wall with transparent vaginal hooks (about 10 hooks/200 μm^2^). Hooks located on low conical vaginal papillae. Vaginal hooks small (< 0.1 mm in length), short and expanded at base; tips pointed and straight to slightly curving away from genital orifice (Fig. 10E, F).

##### Etymology.

The specific epithet “*unidentata*” derived from the Latin words “*unus*” meaning “one” and “*dens*” meaning “tooth”. It referred to a single parietal lamella (or teeth) of the new species.

##### Distribution.

This species is known only from the type locality, Houaphanh, a limestone karst area.

##### Remarks.

Shell variation is evident from specimens from Tam Than Kaisone, about 20 km west of the type locality (Fig. 5H, I; CUMZ 6284, 6285). They are smaller, with a sinuous parietal lamella, and sometimes lack the upper palatal lamella (Table 1). However, only five shells and no living specimens were collected, so we provisionally identifying them as the same species.

#### Perrottetia megadentata

Taxon classificationAnimaliaPulmonataStreptaxidae

Inkhavilay & Panha sp. n.

http://zoobank.org/3DE66E56-8480-4993-91C8-47E885EE2C4D

Figs 1 , 6A, B ; Table 1

##### Type material.

Holotype CUMZ 6286 (Fig. 6A). Measurement: shell height 7.1 mm, shell width 8.2 mm, and with 6 whorls. Paratypes: CUMZ 6287 (31 shells; Fig. 6B), CUMZ 6288 (2 shells), NHMUK (2 shells), NUOL 20160251 (2 shells), SMF (2 shells).

##### Type locality.

The limestone outcrop at Ban Phone Can, Yommalat, Khammouan, Laos (17°31'35.6"N, 105°9'40.7"E)

##### Diagnosis.

The characters distinguishing *Perrottetia megadentata* sp. n. from *Perrottetia daedaleus*, *Perrotettia aquilonaria*, *Perrottetia dugasti* and *Perrottetia mabillei* are a single large parietal lamella, the absence of a palatal lamella absent and the presence of an infra-columellar lamella. The other four species have two parietal lamellae and a palatal lamella. Furthermore, *Perrottetia dugasti* and *Perrotettia aquilonaria* have a smooth shell, slightly depressed spire, and a bifid columellar lamella (Fig. 5A, E). *Perrottetia daedaleus* and *Perrottetia mabillei* have strong transverse ridges over the entire shell, a palatal lamella, and a bifid basal lamella, a columellar lamella is absent in *Perrottetia mabillei* (Fig. 5B), while one basal and one columellar lamella are present in *Perrottetia daedaleus* (Fig. 5C). The new species differs from *Perrottetia unidentata* sp. n. in its ovate shape, smooth shell surface, thicker shell, in the absence of a palatal lamella, and in having infra- and supra-columellar lamellae. The new species is superficially similar to *Perrottetia dermapyrrhosa* Siriboon & Panha, 2013, but is distinguished by having a single and large parietal lamella, and in the absence of a palatal lamella.

##### Description.

Shell oblique-ovate, white and translucent; whorls 6, spire conical, with distinct suture. Shell surface glossy with transverse ridges near suture. Embryonic shell large, about 2½ whorls, with a smooth surface; following whorls regularly coiled. Shell periphery rounded; last whorl axially deflected; two shallow and short longitudinal furrows present. Aperture subcircular, peristome continuous; parietal callus thickened; lip thickened, expanded and reflected; short sinulus present. Apertural dentition with very large and strong sinuous parietal, one large basal lamella located deep inside aperture, one small infracolumellar lamella, one large columellar lamella, and one small supracolumellar lamella. Umbilicus widely open and deep (Fig. 6A, B)

##### Etymology.

The specific epithet “*megadentata*” is derived from the Greek word “*mega*” meaning “large” and the Latin word “*dens*” meaning “tooth”. It referred to the single large parietal lamella of the new species.

##### Distribution.

This species is known only from the type locality in central Laos.

##### Remarks.

To date no living specimens have been collected.

#### Genus Indoartemon

Taxon classificationAnimaliaPulmonataStreptaxidae

Forcart, 1946

Oophana (Indoartemon) Forcart 1946: 215. Benthem Jutting 1954: 95.
Indoartemon —Zilch 1960: 562. Richardson 1988: 223. Schileyko 2000: 776, 777. Siriboon et al. 2014b: 162.

##### Type species.

*Streptaxis eburnea* Pfeiffer, 1861, by original designation.

##### Remarks.

The genus *Indoartemon* can be recognized by the dentition, which consists of one parietal and one palatal lamella (a basal lamella is also present in some species). The penis is long, with a thin penial sheath extending about half of the penis length, through which the vas deferens does not pass. Penial hooks are present (Siriboon et al. 2014b).

Currently, ten species are recognized, of which seven were reported from Indochina south of China and Hainan. Only one species, *Indoartemon tridens* (Möllendorff, 1898) has previously been recorded from Laos (Richardson 1988, Schileyko 2000, Siriboon et al. 2014b); here we describe another.

#### Indoartemon tridens

Taxon classificationAnimaliaPulmonataStreptaxidae

(Möllendorff, 1898)

Figs 1 , 6C

Streptaxis tridens Möllendorff 1898: 67. Type locality: Boloven, Laos [=Boloven Plateau, Paksong, Champasak, Laos]. Gude 1903: 220.
Odontartemon tridens —Kobelt 1905: 94, 95, pl. 58, figs 19, 20.
Indoartemon tridens —Zilch 1961: 85, pl. 5, fig. 15. Richardson 1988: 225. Schileyko 2011: 23.

##### Material examined.

Holotype SMF 108507 (Fig. 6C).

##### Remarks.

Shell oblique-ovate with 5½ whorls, semi-transparent, spire slightly convex, with distinct sutures. Shell surface glossy white with thin growth lines; following whorls regularly coiled. Last whorl axially deflected. Aperture triangular; peristome continuous; lip thickened, little expanded and slightly reflected. Apertural dentition with one large parietal lamella, one palatal lamella, and one small bifid columellar lamella.

Only the type specimen was examined. *Indoartemon tridens* differs from *Indoartemon eburneus*, *Indoartemon prestoni* (Gude, 1903) and *Indoartemon medius* Siriboon & Panha, 2014 from Thailand by having a bifid columellar lamella, an ovate-heliciform shape, its smooth shell surface, narrow umbilicus, and having the left side of penultimate whorl extended beyond the diameter of last whorl. For comparison, *Indoartemon eburneus* and *Indoartemon prestoni* have a less deviated last whorl, transverse ridges on the shell, and a widely open umbilicus; *Indoartemon medius* has an angular penultimate whorl and strong transverse ridges.

#### Indoartemon diodonta

Taxon classificationAnimaliaPulmonataStreptaxidae

Inkhavilay & Panha sp. n.

http://zoobank.org/64F31C73-88D6-4A6B-BD0C-09FE2663E28F

Figs 1 , 6D–F ; Table 1

##### Type material.

Holotype CUMZ 6289 (Fig. 6D). Measurement: shell height 7.5 mm, shell width 8.3 mm, and with 7 whorls. Paratypes: CUMZ 6290 (44 shells; Fig. 6E), NHMUK 20160252 (2 shells), NUOL (2 shells), SMF (2 shells).

##### Other material examined.

Tam Nang Ann, Tha Khek, Khammouan, Laos: CUMZ 6291 (7 shells, Fig. 6F). Tam Xieng Lieb, Tha Khek, Khammouan, Laos: CUMZ 6292 (15 shells).

##### Type locality.

Tam Xang, Tha Khek, Khammouan, Laos, 17°25'44.0"N, 104°51'49.1"E.

##### Diagnosis.

This new species superficially resembles *Indoartemon eburneus* and *Indoartemon prestoni* from Thailand, but it differs in having a much smaller shell, an oblique-heliciform shape, open umbilicus, and the last whorl is strongly deviated from the vertical axis. This species differs from *Indoartemon medius* from Thailand in its smaller shells, angular penultimate whorl and thin transverse ridges. *Indoartemon diodonta* sp. n. differs from *Indoartemon bidens* (Möllendorff, 1883) from Hainan and *Indoartemon tridens* by having fine transverse ridges on the upper periphery, and the last whorl is less deviated from the vertical axis. These two species also have a smooth shell surface and a more strongly deviated last whorl, and a bifid columellar lamella is present in *Indoartemon tridens*.

##### Description.

**Shell.** Shell oblique-heliciform, white and translucent; whorls 6½–7, spire conical, with distinct suture. Shell surface dull, with fine transverse ridges that diminish below the periphery. Embryonic shell large, about 2½ whorls, with smooth surface; following whorls regularly coiled. Last whorl shouldered, axially deflected, and not expanded. Aperture subcircular; peristome continuous, parietal callus thickened; lip thickened, expanded and little reflected. Apertural dentition with one large and strong parietal and one small palatal lamellae. Umbilicus narrow and deep (Fig. 6D–F).

##### Etymology.

The specific epithet “*diodonta*” is derived from the Greek words “*di*” meaning “two” and “*odontos*” meaning “tooth”, referring to the dentition of the new species.

##### Distribution.

This species is known from limestone karst in Khammouan Province, central Laos. The animals can be found at altitudes up to 140 meters above mean sea level.

##### Remarks.

To date no living specimens have been collected.

## Discussion

This study increases the number of streptaxid species recorded from Laos to twelve, three of which are new. Streptaxids occur in both limestone and non-limestone areas in the central and northern parts of Laos. The fauna apparently remains less diverse than that of Thailand and Vietnam (Panha 1996, Hemmen and Hemmen 2001, Siriboon et al. 2013, 2014a, b, Schileyko 2011). The highly modified habitats of southern and some central areas of Laos may harbour a lower species diversity. For example, *Indoartemon tridens* was recorded in 1898 by Möllendorff from its type locality at Boloven plateau, Paksong, Champasak, Laos, but our surveys yielded no specimens collected from this locality.

The species can be separated by geography, shell morphology, and (where available) genital anatomy. Two species from the genus *Haploptychius*; *Haploptychius pellucens* and *Haploptychius porrectus* were described from Laos by Pfeiffer (1863). From our results living and shells specimens of *Haploptychius pellucens* and *Haploptychius porrectus* were collected from nine sampling sites in six provinces such as Louang Namtha, Oudomxay, Louang Phrabang, Xayabouly, Bolikhmaxay and Xieng Khaung. Shell morphology and genitalia anatomy were compared between the two species. The two can be separated by having different shell size and shape, as well as differences in the penial sheath, penial hooks, and vaginal wall. The southernmost population of *Haploptychius pellucens* is particularly small. Most records of *Haploptychius* species are from northern Laos, latitude 18°-21°.

*Perrottetia unidentata* sp. n. and *Perrottetia megadentata* sp. n. are the first two species of the genus recorded in Laos, and are geographically and altitudinally separated. *Perrottetia unidentata* sp. n. occurs in northern Laos close to the Lao-Vietnam border at over 700 m above sea level, while *Perrottetia megadentata* sp. n. occurs far to the south and lower than 200 m above sea level (Fig. 1). The two species can be separated by shell morphology. *Perrotettia* has been collected from central to northern Laos, latitude 18°–22°.

*Indoartemon diodonta* sp. n. is the second species of this genus recorded from Laos after *Indoartemon tridens* (Möllendorff 1898). The new species was found in central Laos, while the first was found in southern Laos, at over 1000 m above sea level. In Laos, *Indoartemon* has now been recorded between latitude 14°–18°.

## Supplementary Material

XML Treatment for Discartemon

XML Treatment for Discartemon discus

XML Treatment for Haploptychius

XML Treatment for Haploptychius pellucens

XML Treatment for Haploptychius porrectus

XML Treatment for Haploptychius fischeri

XML Treatment for Haploptychius blaisei

XML Treatment for Perrottetia

XML Treatment for Perrottetia dugasti

XML Treatment for Perrottetia daedaleus

XML Treatment for Perrottetia aquilonaria

XML Treatment for Perrottetia unidentata

XML Treatment for Perrottetia megadentata

XML Treatment for Indoartemon

XML Treatment for Indoartemon tridens

XML Treatment for Indoartemon diodonta
